# Observations on the Inflammatory Endemic, Incidental to Strangers in the West Indies, from Temperate Climates, Commonly Called Yellow Fever, &c.

**Published:** 1819-10-01

**Authors:** 


*Observations on the Inflammatory Endemic, incidental to Strangers in the West Indies, from temperate Climates, commonly called Yellow Fever, &c.* By Nodes Dickinson, of the College of Surgeons, Staff Surgeon to His Majesty's Forces, Member of the Medical and Chirurgical Society, &c. London, 1819. pp. 216.

*Elements of Medical Logick, illustrated by Practical Proofs and Examples; including a Statement of the Evidence respecting the contagious Nature of the Yellow Fever.* By Sir Gilbert Blane, Bart. Fellow of the Royal Societies of London, Edinburgh, and Gottingen; Member of the Imperial Academy of Sciences of St. Petersburgh, and Physician to the Prince Regent. London, 1819. pp. 219.

There may appear, perhaps, some incongruity in the associating of a book on the yellow fever, with one on Medical Logic; but as the discussion of the nature of that disease forms an important part of the contents of the latter work, we have chosen to bring it in contact with one exclusively devoted to the subject, with the view of exhibiting the contrast of opinions in the two latest writers on this apparently interminable source of disputation. In giving the precedence, on this occasion, to Mr. Dickinson, we have been guided by the convenient mode of analysis which that arrangement affords, in rendering much of what it would be otherwise proper to say on Sir Gilbert Blane's work unnecessary. Like many of their predecessors on this question, the authors before us maintain opinions essentially different, "littora littoribus contraria." Mr. Dickinson refers the origin of the yellow fever uniformly to an endemic source; Sir Gilbert Blane, on the contrary, divides yellow fever into three species, the endemic, the pestilential or malignant epidemic, or typhus icterodes, and the sporadic. The second of these he considers to be the disease which, by its epidemic visitations, has spread death and dismay throughout the West Indies, and in many parts of the United States of America, and of the European Peninsula; and this species he invariably ascribes to the effects of human contagion. But we object *in limine* to this arrangement, on the author's own principle of "the great similarity of this pestilential epidemic, to the endemic and sporadic fevers of the Antilles;" and more especially on the grounds of the community of "another symptom, in which all the three species bear a resemblance to each other;" for we consider the presence of the black vomit in tropical fever, to be the *experimentum crucis* of the existence of real yellow fever.

It must be admitted, that the present advocate of contagion has brought with him into the field an imposing array of argumentative power; and were the points at issue capable of being decided by abstract and analytical reasoning, we might entertain some apprehensions for the safety of the faith which we profess. But we are of opinion, that such speculative weapons are very inoffensive, when directed against the ægis of experience; and that, in the present instance, their chief effect will be shown in the occasion which they afford of keeping up the ball of contention, in a controversy as memorable as any recorded in the history of medicine, whether we regard the talent, the zeal, or, (we are sorry to add) in too many instances, the asperity by which it has been distinguished. Sir Gilbert Blane candidly admits that he has not experience of his own "to decide on the various points of difference" between the endemic or sporadic, and the alleged specific pestilential yellow fevers; but, unfortunately, on those points of difference hinges the whole truth or fallacy of the doctrines in dispute. His opinions are, therefore, necessarily imbued with an hypothetical character, but ill-suited to impress conviction on those who entertain opposite views derived from actual experience in the field of disease. They may be dazzled for a moment by the brilliancy of the language, or confused by the speciousness of the reasoning employed in aid of their conversion; but to such, the disadvantageous suggestion of the preponderance of facts over arguments, perpetually recurs, and effaces any transient impression which the mind may have received from the earnest and persuasive manner in which the author inculcates his opinions. It is true, that some ingenious attempts are made to exalt the value of hear-say evidence, at the expence of close observation; but after mature deliberation on their respective advantages, we are not become proselytes to the doctrine, nor ashamed to express our antiquated prejudices in favour of the latter mode of deduction. Nor, in the consideration of the present question, is it our intention to relinquish the vantage ground, which we believe that we occupy, in being able to say with Æneas, "*Quæque ipse miserima vidi.*"

Were the question before us simply one of contagion or of non-contagion, we should probably have rested satisfied with the refutation under which the contagious creed already labours; for we do not perceive that any additional facts are adduced in the present effort in its favour, which tend to invalidate the overwhelming evidence in proof of the yellow fever being destitute of contagious properties, accumulated by the industry and research of Dr. Bancroft. Mr. Dickinson has, however, promulgated some opinions on the ætiology of yellow fever, which we consider to be of great importance, and to make it imperative on us to inquire somewhat into their legitimacy. It is true, that, unlike the pestilential origin, those tenets exert no prejudicial effect in the treatment of that terrible malady, by inculcating the doctrine (happily now almost obsolete) that debility and putrescence are its essential characteristics, thereby disarming us of the only means which experience has shown to be capable of arresting its rapid career to disorganization and death. The distinction set up is, nevertheless, one of magnitude in a prophylactic view, as strongly influential on an object of paramount importance in tropical hygiene—the selection of situation:—and, in the contingency of human affairs, we know not how soon the period may again arrive, when the fate of multitudes may be involved in the application of this or of a different theory.

Mr. Dickinson expresses his belief that yellow fever is not derived either from human contagion (in which we perfectly concur with him), or from effluvia from the soil; but that it is purely a fever of excitement, the effect in every instance of sudden translation to high temperature and of exposure to insolation, or to both. He observes,

"That a great degree of permanent heat must prevail in those parts of the West Indies, where the elevation of the land is not considerable, is a necessary consequence of proximity to the line.

"An effect of this high temperature upon particular constitutions, unaccustomed to its impressions, is seen in the excitement of yellow fever." P. 6.

"The writer here rejects all occult causes; the introduction of a debilitating poison, whether miasmata or fomites." P. 42.

"The cause immediately productive of the inflammatory endemic in those predisposed by the circumstances of constitution and habit, will be commonly discovered to be an exposure to the direct impression of solar radiation, while unaccustomed to its influence, and unprepared for resistance by the adoption of any sufficient preventive means.

The effects of violent muscular exertion and intoxication, must be noticed similar to, and usually concomitant with, those of high atmospheric temperature." P. 84.

Thus, all atmospheric impressions, except the stimulus of insolation, are rejected as causes of yellow fever: and in order to substantiate the exclusion of effluvia from the soil, which are usually recognized as eminently excitive of that disease, the author enters into a train of reasoning, to show that fever from that cause (as well as from human contagion) displays an essentially different character from that of yellow fever.

"The inflammatory endemic, or yellow fever, here considered, is, however, dissimilar in its real character from yellow fever 'strictly so called," that is, the idiopathic fever of human contagion, or of febrific miasma, the produce of particular soils; nor can it be regarded as a topical phlegmasia, for the general affection of the system precedes the topical determinations which are liable to take place to certain organs." P. 8.

After stating from various authorities, that debility forms the first link in the chain of the phenomena of idiopathic fevers, and constitutes their most conspicuous feature, it is remarked,

"No such appearance of oppression, or of debility in the vital powers as presents itself at the commencement of simple idiopathic fever, takes place in the attack of the disease more expressly treated of here." P. 24.

"The inflammatory endemic is not marked by the stages which characterize a paroxysm of idiopathic fever. The symptoms of the first stage have no existence in it. P. 34.

Yellow fever is thus stated to differ from fever, "strictly so called," in the strength not being impaired in the early part of the disease, and in having no corresponding first or cold stage. We consider these to be very unqualified and startling propositions. How commonly have we found, that in the first hours of the seizure, syncope instantly supervened the change from the recumbent to the erect position; from which we are not aware that any other inference can be deduced, than the existence of extreme oppression, or depression of strength. In some cases, *after great exertion in the sun,* or after a full meal, the patient is struck down at once, as if by a blow, with an instant loss of muscular power, and overwhelming debility, from which he never recovers. Indeed, so conspicuous has commonly been the sudden debility induced, that, as we have had occasion to know, some who combined the greatest talent with unrivalled opportunities of observation, even deemed it to be pathognomonic of real yellow fever. When, indeed, the head is principally and violently affected, so as to induce a condition bordering on phrenitis, there will be unquestionably less appearance of debility, than when the brain is less powerfully excited. Also, when free depletion has been practised, much of the general oppression will be removed, and the strength proportionately increased. But these are contingencies which do not establish the rule contended for by the author. Comparing the existing debility at the onset of the attack, with the degree which is usually observed in the idiopathic fevers of this country, at the same period, (in the reigning epidemic for example), the balance of oppression is greatly on the side of yellow fever. In the former, the individual is not unfrequently capable of leaving his bed during the day, for some time after the seizure, even where the disease eventually proves severe. In the latter, the depression is often so great, as to preclude any other than the horizontal position, without the greatest risk of inducing syncope. Neither do we admit the justness of the observation which denies the existence of a cold stage in yellow fever; and, indeed, it may be considered to be a sufficient refutation of that opinion, to state that the author himself elsewhere allows, that "feelings of heat and slight chilliness alternate for a time." We are not disposed to cavil about relative terms, or to ask for a definition of the limitations of coldness and of a chilliness. It is not pretended that a distinct rigor obtains; but we contend, that a feeling of coldness (transient, it is true, but commensurate in duration with the fleeting character of the other phænomena of yellow fever) is always to be detected.* The truth

* The writer of this article well recollects, that during the existence of an epidemic yellow fever in the early part of his tropical service, he retired to rest in perfect health, and was awakened in the night by a disseems to be, that the morbid actions constituting yellow fever are marked by almost inconceivable rapidity, and the duration of the cold stage is proportionately evanescent; but, generally, not the less real; though, from occuring in the night, it is often unnoticed by the patient, and at so early a period, it is seldom observed by the practitioner.

Mr. Dickinson states, that yellow fever is neither an idiopathic fever, nor yet a phlegmasia. The idea, or rather, we should say, the negation (for it leaves us without an idea) is embarrassing; and the question naturally arises, What is it then?

Having assigned our reasons for believing that the author has failed in establishing a distinctive character between the yellow and idiopathic fevers, generally, we proceed to inquire into the correctness of his exclusion of vegeto-animal effluvia, and other remote causes of the former disease; premising that we believe the difference of habit, and the influence of locality will afford the best solution of the various modifications which the disease affects. In the situation of most of the West India Sea Ports (in which strangers usually undergo their ordeal of yellow fever), the continued form is most frequently observed. But we have often remarked that the same exposure and irregularities exercised in places notoriously abounding with marshy effluvia (as the towns of Point-a-Pitre, Guadaloupe, Fort Royal, and Martinique), have been succeeded by the recurrent character of attack, in individuals possessing the *ratio* of predisposition, which elsewhere has produced the continued form. We shall briefly enumerate some circumstances in the history of yellow fever, which appear to us to be irreconcileable with the simplicity of cause assigned by the author; which, in truth, converts the disease in every instance, into sporadic attacks.

First, it is indisputable, that yellow fever occasionally prevails epidemically in the West Indies, after uncertain intervals of comparative exemption. These visitations admit of no explanation (by us who deny the assumption of imported contagion), except by reference to a temporary aggravation of the endemic causes, depending on peculiar

---

tinct sense of coldness, which was soon succeeded by intense heat, and these alternations continued for some time. The succeeding yellow fever was of that concentrated and dangerous character as to afford little probability of the patient ever entering into a discussion relative to its nature.

atmospheric conditions, which can be recognized only by their effects. For although these epidemic visitations are often coincident with, they are not wholly dependent on, fresh importations of unassimilated strangers. We do not pretend that the introduction of such appropriate pabulum is a matter of indifference to the quantity of sickness induced ; but we are persuaded, that the extent of disease is always in the *compound ratio* of the existing intensity of the remote causes (of which insolation forms but a part), and of the number of susceptible subjects, who may be exposed to their influence; for it is notorious, that in some years, the introduction of strangers may take place with comparative impunity under equal circumstances of exposure, not merely to insolation and inebriety, but to every other exciting cause of yellow fever ; while, at other times, even long residents have not immunity. We have the testimony of M. Humboldt, that predisposition to yellow fever is not absolutely limited to European strangers. That scientific traveller observes, in his Essay on New Spain, that the *change* of *intertropical* residence is sufficient to destroy immunity, even in the natives and long residents. In speaking of this disease at Vera Cruz, he remarks, that the native and long resident inhabitants of that place are exempt; but that the natives of Cuba suffer from fever, when they visit Vera Cruz in August and September; and that, conversely, the natives of Vera Cruz die of yellow fever in Jamaica, Havannah, and in the United States of America; an effect which he imputes, not to insolation, but to some slight variation in the nature of the local miasmata.

The influence of season also, on the intensity and frequency of this disease is very conspicuous. But the high temperature between the tropics being " permanent," if the disease depended wholly on the cause assigned by the author, no part of the year would be exempt from its ravages, which is contrary to experience.

The well-known influence of locality in the production and modification of this disease, militates strongly against the imputed unity of cause. We have often witnessed exposure to insolation and inebriety carried to an extreme degree with impunity, in situations not notoriously insalubrious, by those very individuals who, on a future occasion, have fallen the victims of their own over-weening confidence, by practising the same exposure in places abounding with the remote causes of yellow fever; and in which, indeed, the most temperate and guarded conduct is too insufficient to ensure exemption.

Yellow fever of the most fatal description has been often excited in situations where insolation must hold a very inferior rank; as on board ships at sea, from the effluvia of a foul hold. Every nautical practitioner of any length of servitude in the West Indies, must have seen instances of fever arising from this cause, which ceased only on the hold being cleared.

The identity of cause in the continued and recurrent forms, may be further inferred from their frequent conversion into each other.

Lastly, those writers on the epidemics of North America and Spain, who have also witnessed the West India endemic, concur in the identity of those diseases, however much they may differ concerning their origin; and we hold it to be unnecessary to use any words for the purpose of controverting the opinion which ascribes the ultra-tropical epidemics, solely to insolation and inebriety.

In throwing out these observations, in opposition to the singleness of cause advocated by Mr. Dickinson, it is not our intention, however, to deny its occasional efficiency; but we consider such cases to be purely sporadic; and that they form but an inconsiderable proportion of the yearly amount from other more complex and less tangible exciting causes.

Mr. Dickinson has *entitled* himself an anti-contagionist, and has stated, that during an experience of twenty years in the West Indies, *he met with no instance of contagious fever*. From seeming unwillingness to offend in any quarter, he, however, allows (in adverting to the Bulam controversy) that " perhaps a contagious fever might casually appear in the West India Colonies, as in other parts of the World." We cannot congratulate him on the probable success of his attempt to satisfy the belligerents by this display of the flag of neutrality. He has himself identified the " inflammatory endemic" with the malignant pestilential yellow fever of the contagionists, and has unequivocally declared it to be void of contagion. His position, therefore, has no affinity with the predicament of Garrick between the rival Muses, as depicted by the pencil of our admirable Reynolds;—there can be no contention as to which side of the question he belongs; and we confess that we see no want of courtesy in a competent person withholding admissions decidedly adverse to his whole experience.

It is with reluctance that we have felt compelled to differ with the author on the points which have given rise to the preceding remarks; and we turn from the ungracious task with real satisfaction to the part of the work devoted to "the symptoms and treatment" of the disease, on which we freely bestow our tribute of unqualified applause. The judgment and energy which the author exhibits in his descripton and remedial indications of this violent disease, entitle him to the highest praise; and although we trust that it becomes daily less necessary to insist on the essentially inflammatory nature of yellow fever, and on the advantages of commensurate depletory treatment, we thankfully accept the suffrage of this experienced writer in their favour, and enrol his name with pleasure in the list of those who have eminently contributed to effectuate the abolition of a pernicious practice, founded on an erroneous theory.

We have extended our remarks on Mr. Dickinson's views of yellow fever, so as to leave less space than we originally intended for the "Elements of Medical Logick." But this may be of less importance, as our notice of the latter will be restricted to the section which contains the Dissertation on the contagious nature of that disease, which it is evidently the "end and aim" of the author's work to establish; and, on this topic, our opinions have been greatly anticipated in the former part of this article. Our reasons for believing that the disease in question is not an imported or contagious fever, are founded on no inconsiderable share of personal opportunities of observing its laws and habitudes, in almost every variety of situation, during a long series of inter-tropical movements. It is futile to say that this experience has been limited to "mere exceptions"; unless epidemic yellow fevers, in which scarcely an individual, in a large body of men, has escaped an attack, and which have been characterized by the dingy yellowness of the skin, black vomit, and a high rate of mortality, be considered as "exceptions." That it refers to endemic fevers, we do not deny; but they were endemic fevers in which the rate of mortality has sometimes amounted to one in three of the number attacked; which, on the author's own principles, identifies them with the "pestilential or malignant" disease. We, therefore, protest before hand against the arguments which the author employs in aid of the superiority of hearsay evidence over actual experience. The affinity of the duties of "a judge on the bench," or of "a soldier in a battle," with the labours of an individual cultivating the field of ætiological inquiry, is remote indeed. We cannot better enforce our objections to such loose analogies, than by giving the author's own words, where he adverts to the obscurity in which the history of contagion is involved; "There is here, therefore, the utmost risk in reasoning from analogy; and no inferences can be trusted but those which consist in those pure matters of fact, which constitute each particular case." And were it admissible to reply in the figurative language of which we have complained, we might add, that on the principle contended for, "a fellow that never set a squadron in the field" might arraign the splendid achievements of a Wellington; or a fire side traveller might place himself in the same niche with a Cook, or a Vancouver.

It has been already observed, that the author has engaged pretty extensively in abstract and analogical reasoning in support of the doctrine of contagion. To this species of argument, our reply is, that we conceive the mere list of the names of the anti-contagionists who speak from personal experience,* to be the best, and a perfect answer to any thing which can be advanced on speculation or hearsay. From those names, we could at a glance select many whose talents would not suffer by the comparison with any; and whose experience in yellow fever, individually, would perhaps out weigh that of the opposite party collectively. Indeed, though our author is so thorough a convert to the creed of the latter, the delay for which he thinks it necessary to apologize, would seem to shew that he had felt some reluctance in differing from, or at least in characterizing, as "a deplorable and mischievous delusion," the belief entertained by "so many partisans of respectable talents, and unquestionable good intentions." In the author's Historical Survey of Yellow Fever, we can discover no proof that its epidemic visitations in various parts of the world, have originated in importation or contagion. It is true, that Ligon ascribes the sickness at Barbadoes, in 1647, to importation by "certain ships which had just arrived from long voyages." Its appearance at Lisbon, in 1723, is also explained by stating that it was "probably brought from Brazil:" and the origin at Boston, in 1693, is accounted for by a statement that "it was believed to be brought by the fleet from the West Indies, under Admiral Wheeler, and probably derived from what was imported in the fleet from Siam, a few years before." To these and other alleged importations, is

---

* Bancroft's Sequel. Page 121, and Seq.

added the Bulam statement, labouring as it does under the conviction of error; but how it can be said that "this fever was believed on good grounds to have been brought to Grenada" (alluding to that account), by any one who has read the refutation by facts, we are utterly at a loss to conceive. Indeed, the author here virtually admits the supposed sickly state of the vessel, said to have brought the infection, to be disproved; for he arraigns Dr. Chisholm's "great anxiety to prove that a malignant fever must have previously prevailed in this ship," on the principle that it is well known that typhous infection can be conveyed by men in health. Now, we readily admit that persons confined together, filthy and deprived of air and exercise, may affect others in this way, as has happened in some noted instances of incarceration; and likewise that the same thing may occur in a crowded ship. But that this should happen in a ship not crowded, and especially within the tropics, where free ventilation becomes imperative, we think by no means likely. Likely! did we say? On the contrary, that sailors, whose clothing must be bleached upon their backs by "sun and shower," numberless times before they cross the Atlantic; who may be said to live in the open air by day, and whose duty calls them on deck during the greater part of the night; that men accustomed, like their native oak, to brave all vicissitudes of weather and clime; that such men should become walking pestilences, and carry about their persons infection from one side of the Atlantic to the other, yet themselves appear in perfect health the while, "exceeds the being of belief."

We shall here briefly allude to the two instances of alleged contagious communication at page 159, which have a very imposing appearance. We must remind the author of his quotation from Dr. Cullen, that "there are, in physic, more false facts, than false theories;" for, in the first place, the Carnation was not "a merchant ship," but a brig of war; and though this may not seem very material, yet when we find inaccuracy in one part, we naturally suspect it in another. Not that we mean to question instances of yellow fever having occurred among the crew of the Carnation after their capture, while at sea; for we see nothing in cases of fever supervening on the excitement of an engagement of some hours, and the depression and irregularities consequent upon the capture. If to our question, but were these real cases of yellow fever? it should be answered, yes, for they terminated in black vomiting; we would rejoin, so do sporadic cases induced by over exertion, or any other exciting cause; and, considering that during the short time the vessels were at sea after the capture, there was hardly time for infection (if any existed) to produce its full effect, it is on these grounds far more likely that the fever of the Carnation's men was of the former, than of the latter origin. Respecting the other instance, so little circumstantial, also, is the account, that we do not pretend to form a judgment; but, from our knowledge of navigation, we should be apt to infer, that from the northern degree of latitude which a ship usually traverses in a passage from the West Indies to Europe, the yellow fever must be extinguished long before she could reach the latter shores. That an infectious fever did prevail in that ship during the passage to Brest, may be very probable; and yet it may not have been the yellow fever; and even if the occasional causes, whatever they were, by acting on that peculiarity of habit acquired by even ultra-tropical summer heat, produced symptoms resembling those of the fever of the Antilles, we might still reply to the author, in his own words, "but admitting the symptoms to be ever so similar, it does not follow that they are *identical.*" But it is further possible, that the crew might have been exposed, in some of the ports in the Mediterranean, to the endemic causes of the bilious remittent, which, in its aggravated form, strongly resembles genuine yellow fever. We refer to Dr. Burnett's valuable publication on this point. Hence a person believing the contagious nature of yellow fever, would consider its occuring *after* the capture as a positive proof of its having been thus communicated; overlookiug any other causes of fever, and deceived by the *post hoc, propter hoc* inference, the most fruitful of all sources of error in medical reasoning.

But we will now oppose to what we are told, what we have ourselves seen at the naval depôt, at English harbour, Antigua; where, during the late war, the British navy has experienced an immense loss of her invaluable seamen. In various instances within that period, ships have entered that port for the purpose of repair, with their men in perfect health, and have quitted it after the lapse of a few weeks, with the loss of one fourth of their numbers, and, occasionally, of many more. As a high rate of mortality constitutes the true "pestilential, malignant" disease, its identity with the Antigua epidemics, will not, we presume, be disputed. The causes of this destruction of life and health, are sufficiently manifest on endemic and local principles, but utterly irreconcileable with the existence of contagion. The sickly ships have not been placed in quarantine; and, with unrestrained intercourse, the disease has never been communicated to the crews of any of the numerous vessels which were incessantly entering the port for stores, and other occasions of short detention. At the departure of ships thus afflicted, their decks have been crowded with men labouring under yellow fever in all its stages. If the disease depended on contagion, (more especially of such an active and imperishable nature as to be conveyed from Siam and Bulam to the West Indies, and from thence to North America and Europe, as well as from Brazil to Portugal), it is utterly incredible, that with so many opportunities, it should not have been exported from thence to other places, or have infected the crews of other ships in communication with them; but we have never heard of any occurrence, during that period, which might even induce suspicion that the disease has been thus exported. In our hospital practice we have seen men brought in, afflicted with all degrees, and in all stages of yellow fever, who, from imperious necessity, have been placed among other patients, without having observed the disease communicated to those most contiguous in any instance. With this and other negative evidence before us, we trust that it will not be deemed a mark of unreasonable scepticism, if we pause before we yield our faith to a doctrine thus contravened by hourly experience.

In the fact that yellow fever most commonly prevails in sea port towns, we perfectly concur; because, in tropical countries, in these towns (and generally these alone) it finds proper food to prey on. The ships do not import the disease; but they are the vehicles of susceptible subjects, whose avocations confine them chiefly to the sea ports and their vicinity. This circumstance, added to diminished temperature from increased elevation, is an obvious cause of the great exemption of the interior of those places. In reference to the epidemics of North America and Spain, we may observe that the low situation and other local peculiarities of their sea ports, as well as their dense population, and irregular habits of their inhabitants, are highly favourable to the developement of the disease. The converse of this explains the immunity of the interior of those countries. The exemption of inland places, both within and without the tropics, depends, however, more expressly on their greater altitude, which is equivalent to increase of latitude, and virtually places them beyond the parallel which is compatible with the existence of yellow Fever. M. Humboldt states, that even so near the equator as Vera Cruz, yellow fever cannot exist beyond ten leagues in the interior, on account of the ground rising rapidly to the Westward. Neither did the supposed pestilential disease at Grenada extend to the interior of that island, for the reasons we have assigned; although it is stated to have been carried about to the other islands, and to North America.

The alleged good effects of quarantine and seclusion in the prevention of this disease, (which are chiefly derived from the occurrences at Gibraltar), we are of opinion may be correctly explained without reference to contagion. The troops in health were removed in masses from the town to the Neutral Ground, (where, as Dr. Pym states, "there is a constant and strong breeze,)" out of the range of the operation of the endemic causes. Their consequent exemption has been imputed to the communication with the sick being thus cut off. But their immunity may with greater probability be ascribed to their removal from the local sources of the epidemic. We have no means of judging in what situations the *seclusions* at Gibraltar were performed; but there is reason to believe that the parties were absent from the town during their seclusion. Dr. Pym (an authority which Sir Gilbert Blane will not dispute), observes at page 27 of his Observations on the Bulam Fever, that Colonel Fyers and other officers, with their wives and families, cut off communication with the infected, and escaped the disease. From this it might certainly be inferred that they remained in the midst of the epidemic, and owed their escape simply to seclusion. The sequel throws more light on the subject. It is added, that early in December, some of them, viz. Colonel and Mrs. Darby, and Captain and Mrs. Wilkinson, became "fatigued with their quarantine, and considering the disease to be so far got the better of, as to be safe from its attacks, *returned into town* to their quarters, where they were all, as well as their servants, (with the exception of Colonel Darby, who had been in the West Indies), attacked with the disease, and of which Captain Wilkinson died." Now it is incontestable, that if those persons had not *quitted* the town, and thereby removed themselves from the sphere of operation of the local causes, they could not be said to have *returned* to it. We give this instance of separation merely to shew, that the others may be of a kind which (as well as the quarantine practised at Gibraltar) admits of their advantages being fully explained, independent of the prevention of contagious communication.

The author asks several questions at Page 183, and then replies to them himself. After what has been said, we need not add, that we would answer them very differently.

It is curious, also, to note how differently our author expresses his appreciation of the value of personal observation. With the French commissioners in Spain, it was an advantage that they *had not* seen the epidemic, because it left them, like the "judge on the bench," "divested of prejudice." With Sir Joseph Gilpin, Sir James Fellowes, and Dr. Pym, it is an advantage that they *have* seen the epidemic; for the fact of non-liability to future attacks is thereby attested "under their own eyes, so that there does not seem to be the possibility of a fallacy."

Our experience having led us to form very opposite conclusions, we have considered it our duty to adduce those points on which we differ from the author. With feelings of deference for him, which we cannot extend to the doctrine which he advocates, we have no inclination to recapitulate its inconsistences, many of which we pointed out on a former occasion.* Personally unacquainted with Sir Gilbert Blane, we entertain a high respect for his character, and for his estimable work on the Diseases of Seamen, from which, in former days, we derived much profitable information. On the present occasion we freely concede to him, what we ask for ourselves, credit for purity of motive, and sincerity of intention; and we repeat, that in every instance where we have supported a different opinion, we have expressed that difference with regret.

---

* Review of Bancroft's Sequel, No. 26, Medico-Chirurgical Journal, Monthly Series.

*Additional*

